# MicroRNA preparations from individual monogenean *Gyrodactylus salaris*-a comparison of six commercially available totalRNA extraction kits

**DOI:** 10.1186/1756-0500-4-217

**Published:** 2011-06-29

**Authors:** Bastian Fromm, Philip David Harris, Lutz Bachmann

**Affiliations:** 1 Natural History Museum, University of Oslo, PO Box 1172 Blindern, 0318 Oslo, Norway

## Abstract

**Background:**

Describing and evaluating miRNA inventories with Next Generation Sequencing is a goal of scientists from a wide range of fields. It requires high purity, high quality, and high yield RNA extractions that do not only contain abundant ribosomal RNAs but are also enriched in miRNAs. Here we compare 6 disparate and commercially available totalRNA extraction kits for their suitability for miRNA-preparations from *Gyrodactylus salaris*, an important but small (500 μm in length) monogenean pathogen of Norwegian Atlantic salmon (*Salmo salar*).

**Findings:**

We evaluated 1 salt precipitation method (MasterPure™ Complete RNA Purification Kit, Epicentre), 2 Phenol based extraction methods (mirVana Kit, Ambion, and Trizol Plus Kit, Invitrogen), 1 paramagnetic bead extraction method (RNA Tissue kit, GeneMole) and 2 purification methods based on spin column chromatography using a proprietary resin as separation matrix (Phenol-free Total RNA Purification Kit, Amresco, and ZR MicroPrep Kit, Zymo Research). The quality of the extractions from 1, 10 and 100 individuals, respectively, was assessed in terms of totalRNA yield, RNA integrity, and smallRNA and miRNA yield. The 6 RNA extraction methods yielded considerably different total RNA extracts, with striking differences in low molecular weight RNA yield. The Phenol-free Total RNA Purification Kit (Amresco) showed the highest totalRNA yield, but the best miRNA/totalRNA ratio was obtained with the ZR MicroPrep Kit (Zymo Research). It was not possible to extract electrophoretically detectable miRNAs from *Gyrodactylus salaris *with the RNA Tissue Kit (GeneMole) or the Trizol Plus Kit (Invitrogen).

**Conclusions:**

We present an optimized extraction protocol for single and small numbers of *Gyrodactylus salaris *from infected Atlantic salmon that delivers a totalRNA yield suitable for downstream next generation sequencing analyses of miRNA. Two of the six tested totalRNA kits/methods were not suitable for the extraction of miRNAs from *Gyrodactylus salaris*.

## Background

MicroRNAs (miRNAs) are key regulators of many biological processes in eukaryotes [1]. Besides their investigation through bioinformatical analyses of whole genomes, research focuses on analyses of miRNAs from whole organisms, specific tissues, and/or developmental stages without a fully sequenced and annotated genome at hand [2].

MiRNAs are single-stranded, 22 nucleotide long, noncoding transcripts derived from different genome-encoded hairpin precursors, and regulate gene expression by various mechanisms [3]. First described from *Caenorhabditis elegans *[4] they represent the most recently discovered gene regulators, involved in a broad variety of biological processes including cell proliferation and metabolism [5], developmental timing [6], cell death [7], haematopoiesis [8], neuron development [9], tumorigenesis [10], DNA methylation and chromatin modification [11], and as immune defense against viruses [12]. In evolutionary terms miRNAs are unusual in that they are continuously added to, highly conserved, and rarely lost from metazoan genomes [13,14]. Clearly they are under strong selection, and may therefore represent candidate phylogenetic markers. It may even be possible to reconstruct the miRNA complement of the last common ancestor of all Metazoa [15-17].

Several methods for isolation of totalRNA have been developed, with the focus primarily on high molecular weight RNAs [18]. Commercially available totalRNA kits are affordable, fast, and suitable for RNA extraction from a broad spectrum of samples. Most manufacturers promote their products as suitable for extraction of totalRNA, but frequently it is unclear whether they are equally suitable for smallRNA species, and especially for miRNAs. For extractions of these molecules not only is overall RNA quality and integrity an issue, but also due to their low abundance, yield is of high importance. This is a particular issue if only limited material is available, as in the case of, for example, micro-dissected tissue samples or small invertebrates.

The current study focuses on the ectoparasitic platyhelminth *Gyrodactylus salaris *(Monogenea, Gyrodactylidae). This parasite is responsible for a major epidemic disease of wild salmon in Norway and Russia [19], but gyrodactylids are widespread on teleost fishes and several cause disease in aquaculture [19,20]. Apart from this applied importance, an understanding of the miRNA complement of gyrodactylids will contribute to our understanding of platyhelminth, and early metazoan evolution. However, the individual parasites are only 500 μm in length, presenting a challenge for RNA extraction methodologies. In this study we tested 6 commercially available totalRNA extraction kits for their performance when using as few as 1, 10 and 100 *Gyrodactylus salaris *individuals respectively. Particular emphasis was placed on assessing (i) total RNA yield (ii) RNA integrity (iii) smallRNA yield, and (iv) miRNA yield.

## Methods

### *Gyrodactylus salaris *culture

*Gyrodactylus salaris *were maintained on Atlantic salmon (*Salmo salar*) parr in 500 liter tanks in charcoal-filtered and dechlorinated, continuously running Oslo tap water at 5-6°C. Fish were fed daily on pellet feed (Ewos) and maintained under continuous dim illumination [21].

### Sampling of *Gyrodactylus salaris *specimen for totalRNA analyses

Single infected fish were anaesthetised using 0.1% chlorbutanol and killed by pithing, taking care to avoid contamination with blood. Fins were cut and stored in -20°C ethanol in 2 ml RNAse-free DNA loBind tubes (Eppendorf) until further processing. Using a binocular microscope *Gyrodactylus *were removed from fins using a mounted needle, taking care to avoid contamination with fish tissue. Individual parasites were rinsed with ethanol at -20°C to further remove contaminating fish mucus or epithelial cells. For RNA extraction groups of 1, 10, and 100 *G. salaris *individuals were pooled in 10 μl of -20°C ethanol and immediately processed.

### Ribonucleic acid extraction

Six commercially available kits were compared: MasterPure™ Complete RNA Purification Kit (Epicentre, EPI), Molestrips totalRNA basic for the GeneMole extraction robot (GeneMole, GM), Phenol-free Total RNA Purification Kit (Amresco, AMR), ZR RNA MicroPrep kit (Zymo Research, ZR), mirVana Kit (Ambion, AMB), and the Trizol Plus Kit (Invitrogen, INV).

The selected kits represent four different extraction strategies for total RNA: rapid desalting and precipitation (EPI), solid phase extraction on silica-coated magnetic beads (GM), solid phase extraction on filter membranes (ZR, AMR) and sequential organic and solid phase extractions (AMB and INV). EPI utilises gentle lysis in a SDS-containing buffer with proteinase K whereas GM, AMR, ZR, and AMB kits all employ guanidinium thiocyanate-(GTC) containing chaotropic buffers for the lysis of the tissue/cells and the simultaneous inactivation of RNAses. The INV kit uses Trizol, a GTC-containing chaotropic lysis-buffer premixed with phenol. The EPI kit consecutively precipitates proteins and nucleic acids from the sample, and uses RNAse free DNAse for the subsequent RNA purification. Following the protocols of the GM, AMR, and ZR kits the samples in the lysis buffer (LB) are directly loaded onto the respective binding matrix, whereas the AMB and the INV kits include an acid-phenol-chloroform extraction step prior to the binding of the RNA to a silica-based glass-fiber filter membrane in a spin cartridge. Under high concentrations of salt the RNA binds to the silica-matrices whereas DNA and Proteins flow through the column. The bound RNA is eventually washed from the filters/beads and collected (Table 1).

**Table 1:** Summary of the extraction methods and their chemical principles

manufacturer	totalRNA Extraction Kit	lysis	extraction 1		extraction 2
Ambion	mirVana		organic solvents	acid Phenol	solid-phase
					
Invitrogen	Trizol Plus			Trizol	
			
GeneMole	Molestrips totalRNA basic	Guanidinium-thiocyanat based		beads	
					
Zymo	ZymoReserach RNA miniPrep		solid-phase	column	
Amresco	Phenol-free Total RNA Purification				none
			
Epicentre	MasterPure Complete RNA Purification	SDS based	precipitation, DNAse treatment		

Overview of the chemical principles for the assessed totalRNA extraction methods.

For all extractions we followed kit protocols with the minor modification that all extracted RNA was eluted or resuspended in 100 μl RNAse-free Water (Ambion). The AMB kit protocol offers also a microRNAs only option but this was not followed as the totalRNA was also in the focus of this study and would have been lost. For the ZR Kit the quantitative smallRNA recovery option was used which should deliver smallRNA enriched total RNA with reasonable high mass RNA as well.

All kits were assessed for extractions from 1, 10, and 100 *G. salaris *individuals, respectively. The obtained RNA extracts were heat-denatured for 2 min at 70°C and immediately stored in aliquots in 0.5 μl RNAse-free DNA loBind tubes (Eppendorf) at -80°C until further use.

### Determination of RNA concentrations and RNA integrity

Quantification of totalRNA yield and assessment of integrity was done with the Experion (Bio-Rad laboratories) and 2100 Bioanalyzer (Agilent systems using the Experion RNA HighSense Analysis Kit and the Agilent RNA 6000 Pico Kit respectively. Both systems make use of the micro-fluidic electrophoresis technology for the analysis of biological samples. The Small RNA Kit (Agilent) was used to analyse smallRNA and miRNA content with the 2100 Bioanalyzer (Agilent). The Agilent Small RNA kit offers a fast detection of small RNA with a sensitivity higher than agarose or polyacrylamide gels (for further details see: http://www.genomics.agilent.com/CollectionSubpage.aspx?PageType=Product&SubPageType=ProductDetail&PageID=1647). RNA Quality Indicators (RQI) and RNA integrity numbers (RIN, 1 = degraded, 10 = intact), were derived using the system software. All kits were used following the manufacturers protocols, and quantifications were done in duplicate.

### Verification of ribosomal RNA

To verify that totalRNA preparations contained *Gyrodactylus salaris *RNA all preparations and *Salmo salar *RNA controls were reverse transcribed into cDNA using the iScript Select cDNA Synthesis Kit (Bio-Rad) and a temperature profile of 25°C for 5 min, 42°C for 30 min, and 85° for 5 min. The cDNA was probed for 5S, 18S, and 28S rRNA *Gyrodactylus salaris *using the specific primer pairs 5Sforward: 5'-TCACTCGGCTCACGTGACGA-3', 5Sreverse: 5'-GCCCTTAGCCGCCATTTGCG-3'; 18Sforward: 5'-TGGTTAAACCGCAAACGGCT-3', 18Sreverse: 5'-GTCGTCTGGCAACGGTCCAT-3'; 28Sforward: 5'-CCCAGCACCGAAGCCTACGC-3', 28Sreverse: 5'-AAACCGCTTCGGCCTCCACC-3'. PCR primers were designed from GenBank entries Z72477, Z26942, and AJ542394. The PCR amplification was done using the AmpliTaq Gold^® ^Fast PCR Master Mix (Applied Biosystems) and a protocol consisting of 30 cycles with 94°C for 30s, 60°C for 40s, and 72°C for 60s. Only *Gyrodactylus salaris *preparations yielded positive PCR products that were verified by DNA sequencing, for control preparations from uninfected fish no PCR products were obtained (not shown).

## Results and discussion

### Total RNA yield and integrity

**Figure 1:**
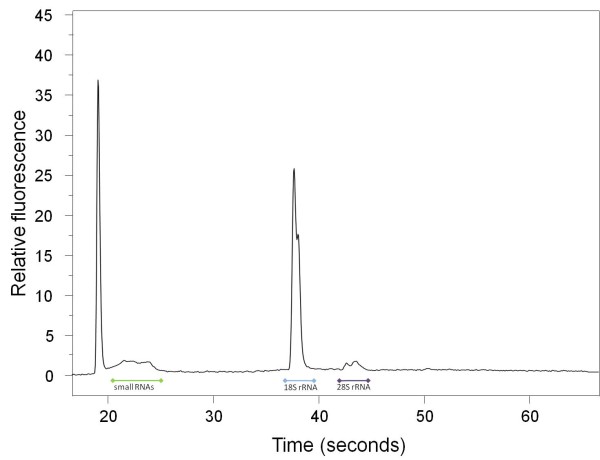
**1 *Gyrodactylus salaris *individual**. Electropherogram (Experion) of totalRNA extracted from 1 *Gyrodactylus salaris *individual using the Phenol-free Total RNA Purification kit from Amresco (AMR). 1% of the extraction was loaded on an Experion RNA HighSens Chip. In *Gyrodactylus salaris *the 28S RNA is probably autocatalytically degraded as described previously for insects [22,23]

**Figure 2:**
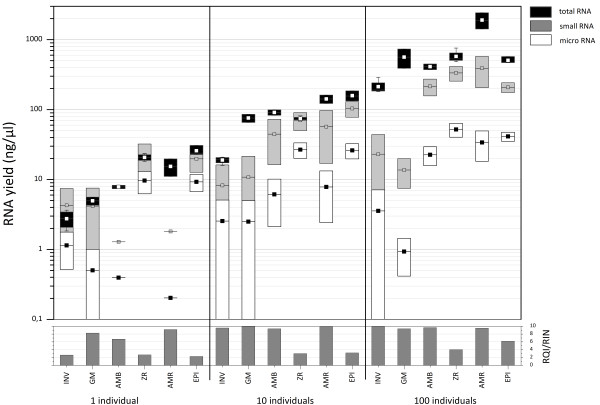
**Boxplot summary of all data**. Semilogarithmic boxplot breakdown of RNA yield for totalRNA, smallRNA and microRNA of all assessed totalRNA extraction kits for 1, 10 and 100 *Gyrodactylus salaris *specimens. RQI/RIN values of the totalRNA as determined by the instrument software following the Experion and 2100 Bioanalyzer electrophoresis systems are depicted at the bottom. Boxes indicate the 25-75% intervals, squares the mean, and whiskers the standard deviation, where this lies outside the 25-75% interval.

The totalRNA yield and integrity of the extractions were compared. The AMR kit was best for extracting high quality totalRNA (RQI, RIN = 10) from single *Gyrodactylus salaris *individuals with a yield of 15.4 ng (Figure 1); the other kits yielded less totalRNA and/or total RNA of lower quality (RQI, RIN < 10). The totalRNA yields ranged between 2.8 ng and 25.8 ng per individual. The respective yields for 10 individuals ranged between 19.0 ng and 158.4 ng, and between 212.8 ng and 190.8 μg for 100 individuals (Table 2 and Figure 2). The overall average yield was 9.7 ng ± 6/individual.

**Table 2:** totalRNA yield in ng

Number of Gyrodactylus salaris individuals	1		10		100		average per individual	
**totalRNA Extraction Kit**	**yield SD**	**Quality SD**	**yield SD**	**quality SD**	**yield SD**	**quality SD**	**yield SD**	**quality SD**

Trizol Plus	2.8 ± 0.8	2.6 ± 1.1	19.0 ± 2.2	9.6 ± 0.4	212.8 ± 50.6	9.9 ± 0.1	2.3 ± 0.4	7.4 ± 4.1
Molestrips totalRNA basic	5.0 ± 0.9	8.3 ± 0.2	75.7 ± 14.2	10.0 ± 0.1	562.7 ± 245.2	9.4 ± 1.3	6.1 ± 1.4	9.2 ± 0.9
mirVana	7.8 ± 0.6	6.7 ± 1.3	91.1 ± 11.6	9.4 ± 0.9	410.9 ± 53.8	9.7 ± 0.5	7.0 ± 2.6	8.6 ± 1.6
ZymoResearch RNA miniprep	20.7 ± 2.4	2.7 ± 0.3	74.0 ± 5.2	3.0 ± 0.1	576.3 ± 124.9	4.0 ± 0.5	11.3 ± 8.2	3.2 ± 0.7
Phenol-free Total RNA Purification	15.4 ± 6.1	9.2 ± 1.2	142.0 ± 27.8	9.9 ± 0.2	1908.5 ± 500.6	9.5 ± 0.9	16.2 ± 2.5	9.5 ± 0.4
MasterPure Complete RNA Purification	25.8 ± 4.3	2.3 ± 0.2	158.4 ± 25.9	3.2 ± 0.5	505.0 ± 59.7	6.2 ± 0.7	15.6 ± 10.4	3.9 ± 2.0

average absolute	12.9 ± 9.2	5.3 ± 3.1	93.4 ± 50.6	7.5 ± 3.4	696.0 ± 608.9	8.1 ± 2.4	**9.7 **± **5.6**	**7.0 **± **2.7**
			
average per individual	12.9 ± 9.2	5.3 ± 3.1	9.3 ± 5.1	7.5 ± 3.4	7.0 ± 6.1	8.1 ± 2.4		

TotalRNA yield and quality of all assessed totalRNA extraction kits for 1, 10 and 100 *Gyrodactylus salaris *specimens. RNA-quality is expressed as RQI/RIN values determined by the software following the Experion and 2100 Bioanalyzer electrophoresis systems respectively. The global average over all methods and sample sizes is depicted in bold.

SD-standard deviation

The yield differences between the kits were highest for extractions from just 1 individual. This may reflect different developmental stages of the respective animals, but may also be due to uncertain estimates due to the sensitivity limitations of the electrophoretic systems. RNA concentrations of less than 50 pg/μl and 100 pg/μl (corresponding to 5 ng and 10 ng in 100 μl) are below the sensitivity limit of the 2100 Bioanalyzer and Experion systems, respectively. Such effects are expected to be less important for the extractions of 10 and 100 individuals.

**Figure 3:**
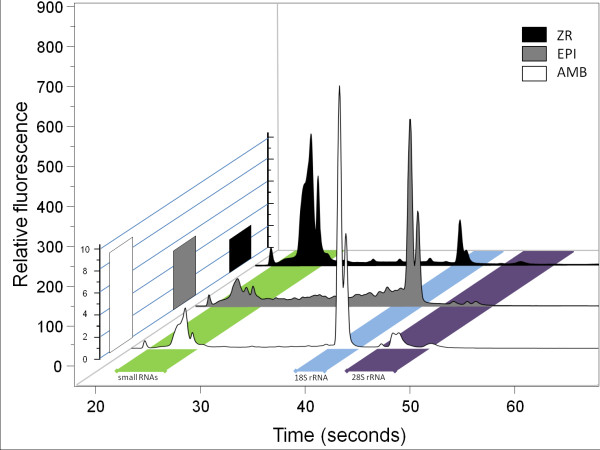
**100 *Gyrodactylus salaris *individuals comparison**. Electropherograms (Experion) of totalRNA preparations from 100 *Gyrodactylus salaris *individuals using the AMB, EPI and ZR kits. 1% of each extraction was loaded on a Experion RNA HighSens Chip. The Z-scale illustrates the RQI/RIN values of the respective preparations. The AMB protocol yields high quality RNA whereas the EPI extract contains a substantial proportion of degraded RNA. The ZR extract is of high quality although the RQI/RIN values are low due to the unusual ratio of smallRNA and 18S/28S RNA.

In general, the quality of totalRNA extractions from single worms was low (RQI/RIN 5.3 ± 3.1) but was much better for 10 (7.5 ± 3.4) and best for 100 (8.1 ± 2.4) individuals. All preparations with the ZR and the EPI kits had relatively low quality indices (2.3-6.2), an indication of increased levels of degradation. The rather poor baseline in the electropherogram shows that, particularly in the EPI preparation, the RNA integrity had suffered from a relatively long extraction time including DNAse treatment. For the ZR kit, however, the low RQI/RIN values seem to have a different explanation. The protocol efficiently extracts small RNAs and the high molecular weight RNAs contribute comparatively less to the totalRNA. Although there is no noise indicating RNA degradation (Figure 3), the distribution of RNA seems to have been misinterpreted by the analysis algorithm as an indication of poor quality.

Interestingly the assessed RNA extraction kits did not only show differences in yield per individual, they also differed significantly in yield per individual between the extractions using 1, 10, and 100 individuals, respectively. This is unlikely to be due to exceeding the extraction limits of the respective kits, and it is also unlikely - at least for the EPI kit, as it is a precipitation kit only-to be a consequence of the binding capacity of the corresponding binding matrix.

**Figure 4:**
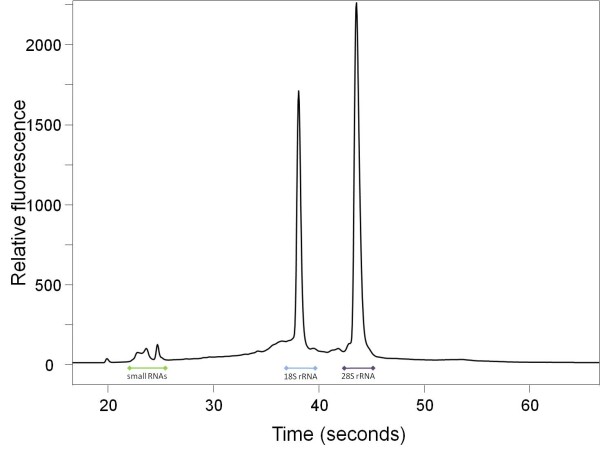
***Salmo salar *RNA**. Electropherogram (Experion) of totalRNA extracted from uninfected fish-tissue prepared with AMR. 1% of the extraction was loaded on an Experion RNA HighSens Chip. The ranges of the 18S, 28S, and small RNA species are indicated.

The electrophoretic profile of all *Gyrodactylus *preparations with a major 18S rRNA peak as the most prominent signal was different from the "usual" totalRNA preparation profile with a high 28S rRNA signal, a less prominent 18S rRNA signal, and a rather low signal from the smallRNAs. A very similar pattern has been reported from insect RNA preparations. For several insects it has been shown to be a result of heat- and chemical-dissociable 28S rRNA species, which autocatalytically degrade into two 18S rRNA pieces [22,23]. Similar processes may also affect the 28S rRNA of *Gyrodactylus*. In our study this peculiarity of *Gyrodactylus *28S rRNA allows us to detect sample contamination with fish RNA. As the Atlantic salmon 28S rRNA does not show this property (Figure 4), a very prominent 28S rRNA peak would point to sample contamination. No such contamination has been observed in any *Gyrodactylus *preparation.

### Small and microRNA yield

As expected the yield of smallRNA of the assessed kits varied as the totalRNA yield: for 1 individual the values ranged between 1.3 ng and 22.3 ng, between 8.2 ng and 103.4 ng for 10, and between 13.6 ng and 389.3 ng for 100 individuals (see Table 3). The overall average yield of smallRNAs was 5.3 ng/individual. The smallRNA fraction includes the microRNAs, and therefore it was no surprise that the microRNA yield differed almost proportionally to the smallRNA yield. The respective values ranged between 0.2 ng and 9.6 ng for 1, between 2.5 ng and 26.6 ng for 10, and between 0.9 ng and 51.6 ng for 100 individuals (see Figure 2). The overall average yield of microRNAs was 1.7 ng/individual. The best microRNA to smallRNA ratio (with the highest absolute microRNA value of 51.6 ng) was achieved with the ZR kit for 100 individuals. In contrast, only very small amounts of either smallRNAs or microRNAs could be detected in extracts from the GM and the INV kits.

**Table 3:** small/miRNA yield in ng

Number of *Gyrodactylus salaris *individuals	1		10		100		average per individual	
**totalRNA Extraction Kit**	**smallRNA SD**	**miRNA SD**	**smallRNA SD**	**miRNA SD**	**smallRNA SD**	**miRNA SD**	**smallRNA SD**	**miRNA SD**

Trizol Plus	4.3 ± 4.5	1.1 ± 0.9	8.2 ± 11.2	2.5 ± 3.6	22.9 ± 29.6	3.5 ± 5.0	1.8 ± 2.2	0.5 ± 0.6
Molestrips totalRNA basic	4.2 ± 4.7	0.5 ± 0.7	10.8 ± 15.1	2.5 ± 3.5	13.6 ± 8.8	0.9 ± 0.7	1.8 ± 2.1	0.2 ± 0.2
mirVana	1.3 ± n.a.	0.4 ± n.a.	44.4 ± 39.9	6.1 ± 5.6	214.2 ± 81.7	22.4 ± 9.6	2.6 ± 1.6	0.4 ± 0.2
ZymoResearch RNA miniprep	22.3 ± 13.8	9.6 ± 4.8	70.0 ± 28.5	26.6 ± 9.4	333.4 ± 112.0	51.6 ± 16.4	10.9 ± 10.1	4.3 ± 4.7
Phenol-free Total RNA Purification	1.8 ± n.a.	0.2 ± n.a.	56.9 ± 56.6	7.8 ± 7.6	389.3 ± 261.0	33.7 ± 22.0	3.8 ± 2.0	0.4 ± 0.3
MasterPure Complete RNA Purification	19.7 ± 10.0	9.2 ± 3.6	103.4 ± 36.5	25.9 ± 9.0	207.7 ± 47.1	41.1 ± 8.7	10.7 ± 8.8	4.1 ± 4.6

average absolute	8.9 ± 9.5	3.5 ± 4.6	49.0 ± 36.4	11.9 ± 11.3	196.9 ± 154.9	25.6 ± 20.5	**5.3 ± 4.3**	**1.7 ± 2.0**
			
average per individual	9.7 ± 9.2	3.9 ± 4.4	4.9 ± 3.6	1.2 ± 1.1	2.0 ± 1.5	0.3 ± 0.2		

SmallRNA and microRNA yield of all assessed totalRNA extraction kits for 1, 10 and 100 *Gyrodactylus salaris *specimen. The global average over all methods and sample sizes is depicted in bold.

SD-standard deviation

Meaningful assessment of the yield of smallRNAs depended on the quality of the sample, as an electropherogram of degraded RNA may resemble that of samples with a high smallRNA content. Given the low RQI/RIN values we consider the relatively large smallRNA fraction obtained with the EPI kit as mainly consisting of degraded high molecular weight RNA.

Summarizing the data gathered in this study an average *Gyrodactylus salaris *individual yields 9.7 ng totalRNA out of which 5.3 ng are small RNAs, including 1.7 ng microRNAs.

## Conclusions

We have demonstrated successful extraction of high quality RNA from a single *Gyrodactylus salaris *individual, a small monogenean ectoparasite with a body length of 500 μm only. Careful choice of totalRNA extraction kit is crucial, and in our comparison, the best results for totalRNA containing smallRNAs were obtained with the Phenol-free Total RNA Purification Kit (Amresco). However the highest amount of miRNAs with a yield of 9.6 ng for one individual could be extracted using the ZR RNA MicroPrep kit (Zymo Research), which is also the only kit that allows elution with a volume as little as 6 μl, one order of magnitude smaller than for the other kits and recommended as input for some downstream NGS applications (8 μl, ScriptMiner™ Small RNA-Seq Library Preparation Kit, Lit. # 316 · 11/2010, Cambio).

The amount of totalRNA extracted in this study suggests that a sample size of 100 *Gyrodactylus salaris *individuals is sufficient for a microRNAs sequencing run with an Illumina Genome Analyzer 2 (1-5 μg, ScriptMiner™ Small RNA-Seq Library Preparation Kit, Lit. # 316 · 11/2010, Cambio), making such genomic and transcriptomic analyses feasible with manageable numbers of these pathogenic fish parasites.

Other totalRNA extractions kits such as the Molestrips totalRNA basic for the GeneMole extraction robot (GeneMole) and the Trizol Plus Kit (Invitrogen) were found to be inappropriate for extracting small RNA and microRNA from rather small samples. This may be of particular interest as kits from the Trizol (Invitrogen) family have frequently been used as the standard extraction method in recent microRNAs studies (e.g., [17]).

The successful extraction of suitable amounts of RNA, especially miRNAs of reasonable quality offers an interesting novel tool for assessing the miRNA complement of single small sized parasites including those of different developmental stages.

## Competing interests

The authors declare that they have no competing interests.

## Authors' contributions

BF conceived the study, designed and carried out all the experiments and drafted the manuscript. LB and PDH participated in the design of the study and contributed to drafting the manuscript. All authors have read and approved the final version of the manuscript.
